# Publications of systematic review and meta-analysis in the indexed anesthesia journals: a 10-year bibliometric analysis

**DOI:** 10.3389/fmed.2025.1523630

**Published:** 2025-05-07

**Authors:** Zhi-yao Zou, Xiao-jing Huang, Jin-rui Song, Yun-tai Yao

**Affiliations:** ^1^Department of Anesthesiology, Fuwai Yunnan Hospital, Chinese Academy of Medical Sciences/Affiliated Cardiovascular Hospital of Kunming Medical University, Kunming, Yunnan, China; ^2^Department of Anesthesiology, Fuwai Hospital, National Center for Cardiovascular Diseases, Peking Union Medical College and Chinese Academy of Medical Sciences, Beijing, China

**Keywords:** anesthesia journal, systematic review, meta-analysis, bibliometrics, citation

## Abstract

**Background:**

Anesthesiology research is growing at a rapid pace. It is essential to understand the scope and trends over time to identify gaps and future areas for growth. Systematic reviews and meta-analyses (SRMA) are summaries of the best available evidence to address a specific research question via a comprehensive literature search, in-depth analyses, and synthesis of results. High-quality SRMA are increasingly used and play an essential role in medical research.

**Objective:**

We aimed to explore the trends of SRMA in indexed anesthesia journals.

**Methods:**

SRMA published in indexed anesthesia journals from 2013 to 2023 were retrieved from the Web of Science database. Data were presented via descriptive statistics. We used CiteSpace 6.1.R6 to analyze countries, institutions, journals, authors, and keywords through visual maps to explore the research hotspots and trends. The journal’s Journal Citation Reports partition, impact factor, annual publications, journals H-index, and a number of highly-cited papers were calculated in the WoS database.

**Results:**

A total of 34 indexed anesthesia journals and 3,004 SRMA were included. The year 2021 was the year with the most SRMA (385/3,004). Out of the 3,004 SRMAs, 36 (0.03%) were highly cited papers, and 22 of the 36 highly cited papers focused on “pain management.” BRITISH JOURNAL OF ANAESTHESIA had the highest 5-year impact factor (9.6) in 2022 Journal Citation Reports, the most significant number of publications (268/3,004), the highest total number of citations (13,173/86,145), and the most significant number of SRMAs cited more than 100 (36/160). ANAESTHESIA achieved the highest impact factor in the 2022 Journal Citation Reports (10.7) and the highest average annual citations (58.82). PAIN had the highest number of highly cited papers (15/36). The United States of America was the most productive country, with 823/3,004 SRMAs. University Toronto had the highest number of publications (245/3,004). The most frequent of keywords was the topic “Pain Management” (1,622/29.1%).

**Conclusion:**

This present study would be valuable to practitioners, academics, researchers, and students in understanding the dynamics of progress in anesthesiology.

## 1 Introduction

Anesthesiology research is essential for understanding anesthesia and related fields of medicine (1). The number of articles published by an institution or country in indexed anesthesia journals indicate its contribution to creating new knowledge in anesthesiology (2, 3). However, the results of individual studies are often insufficient to provide confident answers, as their results are not consistently reproducible. A meta-analysis is a statistical method for combining the results of different studies on the same topic, and it may resolve conflicts among studies. A systematic review is a literature review focused on a single question that attempts to identify, evaluate, select, and synthesize all high-quality research evidence related to that question (4). High-quality systematic reviews and meta-analyses (SRMA) are increasingly used and play an essential role in medical research. The quality of meta-analyses published in indexed anesthesia journals was moderate to high, with statistically significant improvements over time (5).

In recent decades, the number of published SRMA has grown exponentially. According to PubMed, 435 SRMA were published in 1995 compared to 20,774 in 2017, representing a growth rate of approximately 4,676% (6). Such growth has increased overlap and redundancy among research topics, with only around 3% of SRMA estimated to be methodologically sound and non-redundant or to provide useful clinical information (7).

Bibliometric analyses offer one approach for identifying critical studies. They are a type of literature analysis comprising a collection of quantitative and statistical tools for evaluating the quality and impact of the literature associated with a certain topic or field (8). There has been an increasing number of bibliometric analyses in anesthesiology in recent years (5, 9, 10). These documents systematically revealed the productivity and collaborations of institutions, journals, and countries, making monitoring the development of a specific field possible. This present study would be valuable to practitioners, academics, researchers, and students in understanding the dynamics of progress in anesthesiology. Thus, we aimed to explore the trends of SRMA in indexed anesthesia journals from 2013 to 2023 according to bibliometric methods.

## 2 Methods

Journals related to anesthesiology were selected from the ‘anesthesiology’ category in 2022 Journal Citation Reports (JCR) established by the Institute for Scientific Information (ISI): https://jcr.clarivate.com/jcr/browse-journals. A total of 65 journals related to anesthesiology were selected. We included only the journals with an ISSN (print) number in the Science Citation Index Expanded (SCIE) of the WoS Core Collection. As a result, one journal without an ISSN (print) number and 30 non-SCIE journals were excluded from the study. WoS Core Collection (Clarivate Analytics) is a research platform that provides a substantial bibliographic database through the Science Citation Index Expanded (SCIE).

The Web of Science database was searched to retrieve SRMA publications in indexed anesthesia journals between 2013 and 2023. Data were presented via descriptive statistics. We used CiteSpace 6.1.R6 to analyze countries, institutions, journals, authors, and keywords through visual maps to explore the research hotspots and trends. The journal’s JCR partition, impact factor (IF), annual publications, journal H-index, and a number of highly-cited papers were calculated in the WoS database. We used CiteSpace 6.1.R6 to analyze keywords, and the type of each topic was manually coded through two authors.

A computerized literature search was conducted in the WoS database from January 1st, 2013, to December 31st, 2023. The titles of the 34 journals were used to perform searches in WoS, TS = (“meta analyses” OR “meta analyses” OR “systematic review”), language = “English,” type = “article or review,” search strategy in Supplementary 1.

## 3 Statistical analyses

Data were presented via descriptive statistics.

## 4 Results

### 4.1 Journals JCR partition and impact factor

Finally, 34 journals were included in this study, according to the 2022 JCR established by the ISI: Q1 (8;24.00%), Q2 (9;26.00%), Q3 (8;24.00%), Q4 (9;26.00%). The 34 included journals have IF and 5y-IF. The IF of the 34 journals in 2022 ranged from 0.4 to 10.7. Among these were 8 (24.00%) journals with an IF greater than 5. The top 10 journals with the highest IF: ANAESTHESIA: 10.7, BRIT J ANAESTH: 9.8, ANESTHESIOLOGY: 8.8, PAIN: 7.4, J CLIN ANESTH: 6.7, ANESTH. ANALG: 5.9, ANAESTH CRIT CARE PA: 5.5, REGION ANESTH PAIN M: 5.1, BEST PRAC RES-CL ANA: 4.8, CAN J ANEST: 4.2, as listed in Table 1.

**Table 1:** Anesthesia journals.

No	Journal name	Abbreviation	2022 impact factor	5 years impact factor	Journal citation reports partition
1	Anaesthesia	ANAESTHESIA	10.7	8.4	Q1
2	British Journal of Anaesthesia	BRIT J ANAESTH	9.8	9.6	Q1
3	Anesthesiology	ANESTHESIOLOGY	8.8	8.4	Q1
4	Pain	PAIN	7.4	7.7	Q1
5	Journal of Clinical Anesthesia	J CLIN ANESTH	6.7	6.2	Q1
6	Anesthesia and Analgesia	ANESTH ANALG	5.9	5.7	Q1
7	Anaesthesia Critical Care & Pain Medicine	ANAESTH CRIT CARE PA	5.5	4.5	Q1
8	Regional Anesthesia and Pain Medicine	REGION ANESTH PAIN M	5.1	5.7	Q1
9	Best Practice & Research-Clinical Anaesthesiology	BEST PRAC RES-CL ANA	4.8	4.6	Q2
10	Canadian Journal of Anesthesia-Journal Canadien D Anesthesie	CAN J ANESTH	4.2	4.2	Q2
11	Journal of Neurosurgical Anesthesiology	J NEUROSURG ANESTH	3.7	2.9	Q2
12	European Journal of Anaesthesiology	EUR J ANAESTH	3.6	4.4	Q2
13	European Journal of Pain	EUR J PAIN	3.6	3.9	Q2
14	Minerva Anestesiologica	MINERVA ANESTESIOL	3.2	2.9	Q2
15	Pain Medicine	PAIN MED	3.1	3.4	Q2
16	Korean Journal of Anesthesiology	KOREAN J ANESTHESIOL	2.9	4.8	Q2
17	Clinical Journal of Pain	CLIN J PAIN	2.9	3.8	Q2
18	International Journal of Obstetric Anesthesia	INT J OBSTET ANESTH	2.8	2.6	Q3
19	Journal of Anesthesia	J ANESTH	2.8	2.5	Q3
20	Journal of Cardiothoracic and Vascular Anesthesia	J CARDIOTHOR VASC AN	2.8	2.5	Q3
21	Perioperative Medicine	PERIOPER MED-LONDON	2.6	3.2	Q3
22	Pain Practice	PAIN PRACT	2.6	2.9	Q3
23	Current Opinion in Anesthesiology	CURR OPIN ANESTHESIO	2.5	2.9	Q3
24	BMC Anesthesiology	BMC ANESTHESIOL	2.2	2.6	Q3
25	Journal of Clinical Monitoring and Computing	J CLIN MONIT COMPUT	2.2	2.1	Q3
26	Acta Anaesthesiologica Scandinavica	ACTA ANAESTH SCAND	2.1	2.1	Q4
27	Pediatric Anesthesia	PEDIATR ANESTH	1.7	2.3	Q4
28	Anaesthesia and Intensive Care	ANAESTH INTENS CARE	1.5	1.9	Q4
29	Brazilian Journal of Anesthesiology	BRAZ J ANESTHESIOL	1.3	1.3	Q4
30	Anaesthesiologie	ANAESTHESIOLOGIE	1.1	1.1	Q4
31	Schmerz	SCHMERZ	1	0.9	Q4
32	Revista Brasileira De Anestesiologia	REV BRAS ANESTESIOL	1	1.1	Q4
33	Anasthesiologie & Intensivmedizin	ANASTH INTENSIVMED	0.7	0.5	Q4
34	Anasthesiologie Intensivmedizin Notfallmedizin Schmerztherapie	ANASTH INTENSIV NOTF	0.4	0.5	Q4

The average 5y-IF of the 34 journals in 2022 ranged from 0.5 to 9.6. Among these were 7 (21.00%) journals with 5y-IF greater than 5. The top 10 journals with the highest average 5y-IF: BRIT J ANAESTH: 9.6, ANAESTHESIA: 8.4, ANESTHESIOLOGY: 8.4, PAIN: 7.7, J CLIN ANESTH: 6.2, ANESTH ANALG: 5.7, REGION ANESTH PAIN M: 5.7, KOREAN J ANESTHESIOL: 4.8, BEST PRAC RES-CL ANA: 4.6, ANAESTH CRIT CARE PA: 4.5, as listed in Table 1.

### 4.2 H-index of journals

The H-index of each journal ranged from 0 to 62. The top 10 journals with H-index SRMAs: BRIT J ANAESTH: 62, PAIN: 62, ANESTH ANALG: 48, ANAESTHESIA: 44, EUR J PAIN: 39, PAIN MED: 36, ANESTHESIOLOGY: 34, ACTA ANAESTH SCAND: 30, J CLIN ANESTH: 30, REGION ANESTH PAIN M: 29, CAN J ANESTH: 29, J CARDIOTHOR VASC AN: 29, as listed in Table 2.

**Table 2:** Publications number, journal citations and H-index, and highly cited papers.

		Publications number												Journal citations				
No	Journal	2013	2014	2015	2016	2017	2018	2019	2020	2021	2022	2023	Total	Total citations	Average/year	Citations ≥ 100	H-index	Highly cited
1	ANAESTHESIA	2	14	11	12	13	17	20	23	17	9	12	150/4.99%	5,818	38.79	9	44	4/11.11%
2	BRIT J ANAESTH	21	16	17	17	23	36	19	19	34	41	25	268/8.92%	13,173	49.15	36	62	9/25.00%
3	ANESTHESIOLOGY	9	4	8	8	9	1	4	4	8	4	2	61/2.03%	3,588	58.82	14	34	1/2.78%
4	PAIN	20	16	17	17	10	16	18	23	26	33	30	226/7.52%	12,991	57.48	33	62	15/41.67%
5	J CLIN ANESTH	2	3	3	19	21	20	16	28	28	19	15	174/5.79%	3,223	18.52	1	30	1/2.78%
6	ANESTH ANALG	12	13	15	23	31	29	26	18	16	16	20	219/7.29%	8,034	36.68	15	48	2/5.56%
7	ANAESTH CRIT CARE PA	0	0	0	1	2	2	4	6	8	6	7	36/1.20%	439	12.19	0	12	0
8	REGION ANESTH PAIN M	2	1	3	9	7	5	8	13	14	11	7	80/2.66%	2,305	28.81	3	29	0
9	BEST PRAC RES-CL ANA	0	0	0	0	0	0	0	0	1	1	2	4/0.13%	54	13.5	0	2	0
10	CAN J ANESTH	4	10	9	6	5	12	15	15	13	10	19	118/3.93%	2,323	19.69	2	29	0
11	J NEUROSURG ANESTH	1	1	1	2	2	2	2	1	5	6	7	30/1.00%	514	17.13	0	13	0
12	EUR J ANAESTH	2	3	5	9	5	9	4	13	15	13	8	86/2.86%	2,302	26.77	3	24	2/5.56%
13	EUR J PAIN	8	8	6	13	11	10	15	28	21	21	11	152/5.06%	5,532	36.39	16	39	0
14	MINERVA ANESTESIOL	8	10	11	12	14	11	12	12	12	13	16	131/4.36%	2,182	16.66	2	27	0
15	PAIN MED	10	19	7	10	10	10	21	49	50	32	9	227/7.56%	4,464	19.67	6	36	0
16	KOREAN J ANESTHESIOL	0	0	0	0	0	0	1	0	8	7	3	19/0.63%	183	9.63	1	6	0
17	CLIN J PAIN	11	8	8	14	15	22	18	15	18	14	17	160/5.33%	4,575	28.59	8	37	1/2.78%
18	INT J OBSTET ANESTH	3	0	3	2	2	2	7	6	2	3	3	33/1.10%	551	16.7	0	16	0
19	J ANESTH	2	7	4	4	6	2	3	6	11	8	10	63/2.10%	834	13.24	0	16	0
20	J CARDIOTHOR VASC AN	8	11	8	11	13	15	9	12	14	16	18	135/4.49%	2,734	20.25	2	29	0
21	PERIOPER MED-LONDON	0	0	0	0	2	5	2	6	9	2	7	33/1.10%	336	10.18	0	10	0
22	PAIN PRACT	4	11	5	10	10	12	9	14	11	10	18	114/3.79%	2,405	21.1	3	27	0
23	CURR OPIN ANESTHESIO	3	1	1	3	8	2	8	2	2	6	8	44/1.46%	478	10.86	0	12	0
24	BMC ANESTHESIOL	1	5	8	6	7	12	14	26	13	10	29	131/4.36%	1,794	13.69	0	23	0
25	J CLIN MONIT COMPUT	0	0	0	3	5	1	0	5	2	4	6	26/0.87%	430	16.54	0	11	0
26	ACTA ANAESTH SCAND	6	12	11	9	7	14	27	17	18	18	25	164/5.46%	2,820	17.2	4	30	1/2.78%
27	PEDIATR ANESTH	3	9	8	7	4	4	4	8	7	6	8	68/2.26%	1,374	20.21	2	23	0
28	ANAESTH INTENS CARE	1	0	3	3	3	2	0	2	1	0	0	15/0.50%	265	17.67	0	7	0
29	BRAZ J ANESTHESIOL	0	0	0	0	0	0	0	0	1	6	13	20/0.67%	69	3.45	0	4	0
30	ANAESTHESIOLOGIE	0	0	0	0	0	0	0	0	0	1	0	1/0.03%	1	1	0	1	0
31	SCHMERZ	0	0	0	2	0	0	0	1	0	0	0	3/0.10%	130	43.33	0	2	0
32	REV BRAS ANESTESIOL	2	0	3	3	4	0	0	0	0	0	0	12/0.40%	224	18.67	0	10	0
33	ANASTH INTENSIVMED	0	0	0	0	0	0	0	0	0	1	0	1/0.03%	0	0	0	0	0
34	ANASTH INTENSIV NOTF	0	0	0	0	0	0	0	0	0	0	0	0	0	0	0	0	0
	Total	145	182	175	235	249	273	286	372	385	347	355	3,004	86,145	732.56	160	755	36

### 4.3 Country

The 3,004 SRMAs were published across 89 different countries, and the total number of SRMAs published by each country ranged from 1 to 823. The top 10 countries with the highest number of SRMAs: the United States (USA) (823/3, 004; 27.40%), Canada (513/3, 004; 15.35%), United Kingdom (461/3, 004; 15.35%), China (445/3, 004; 15.15%), Australia (310/3, 004; 10.32%), Denmark (247/3, 004; 8.22%), Germany (214/3, 004; 7.12%), Italy (210/3, 004; 6.99%), Netherlands (204/3, 004; 6.79%), Switzerland (141/3, 004; 4.69%), as listed in Supplementary 2.

### 4.4 Institutions

The 3,004 SRMAs were published across 465 different institutions, and the total number of SRMAs published by each institution ranged from 2 to 245. The top 10 institutions with the highest number of SRMAs: University (Univ) Toronto (Canada, 245), Univ Copenhagen (Denmark, 92), McMaster Univ (Canada, 87), Univ Ottawa (Canada, 85), Copenhagen Univ Hosp (Denmark, 68), Stanford Univ (USA, 68), Univ Sydney (Australia, 68), Monash Univ (Australia, 65), Univ Washington (USA, 59), Kings Coll London (United Kingdom, 57). The 10 institutions were from 5 different countries: Canada (3, 30%), Denmark (2, 20%), USA (2, 20%), Australia (2, 20%), United Kingdom (1, 10%), as listed in Supplementary 3.

### 4.5 Total number of publications

**Figure 1:**
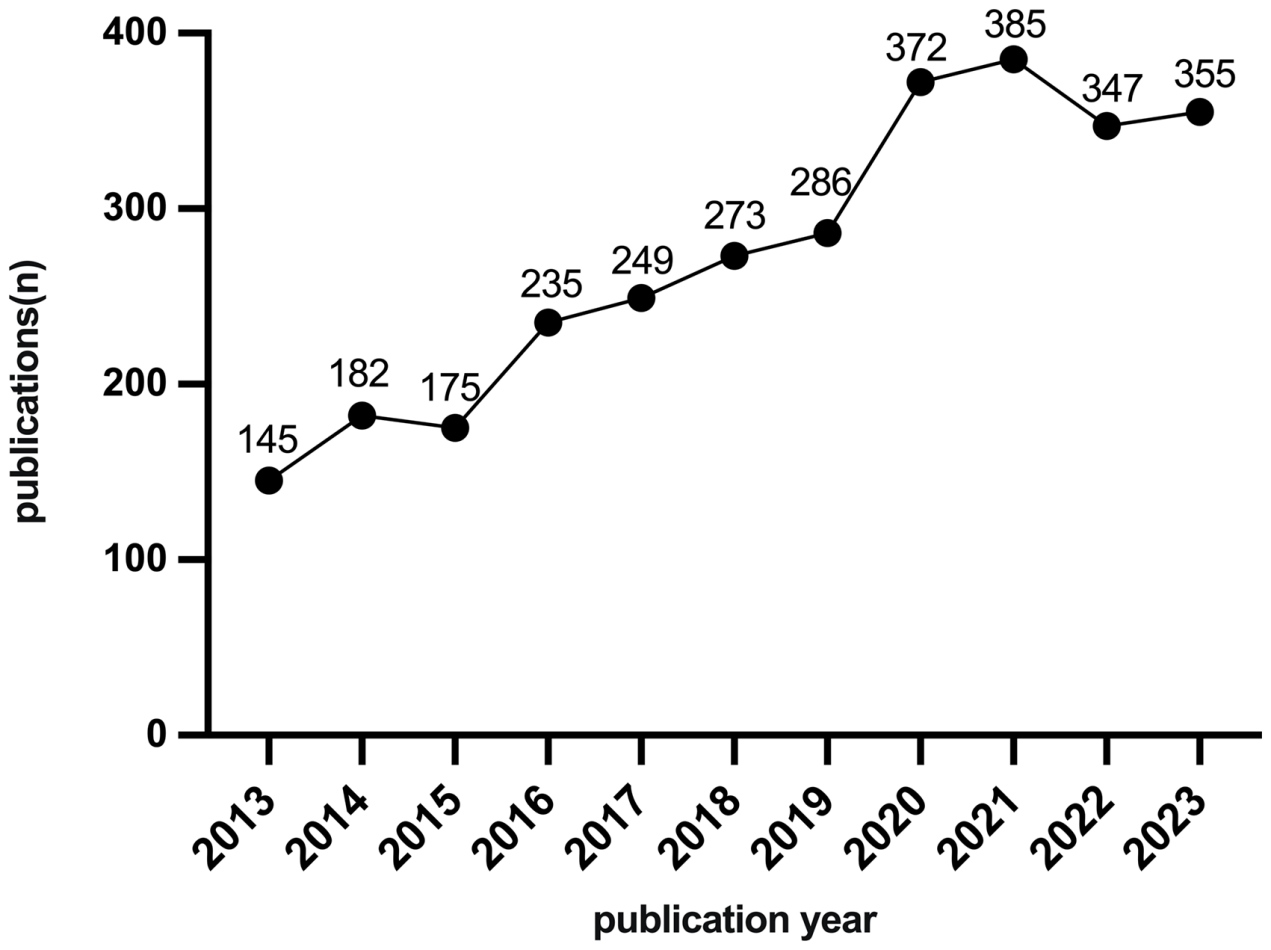
The annual number of systematic reviews and meta-analyses articles in indexed anesthesia journals.

A total of 3,004 SRMAs were published in 34 selected journals from 2013 to 2023 worldwide. Of these, 2,398 were “Review SRMAs” and 606 were “SRMAs,” with an annual range from 145 to 385. 2013: 145/4.83%, 2014: 182/6.06%, 2015: 175/5.83%, 2016: 235/7.82%, 2017: 249/8.29%, 2018: 273/9.09%, 2019: 286/9.52%, 2020: 272/12.38%, 2021: 385/12.82%, 2022: 347/11.55%, 2023: 355/11.82%, as listed in Table 2 and show in Figure 1.

### 4.6 Total number of publications per journal

**Figure 2:**
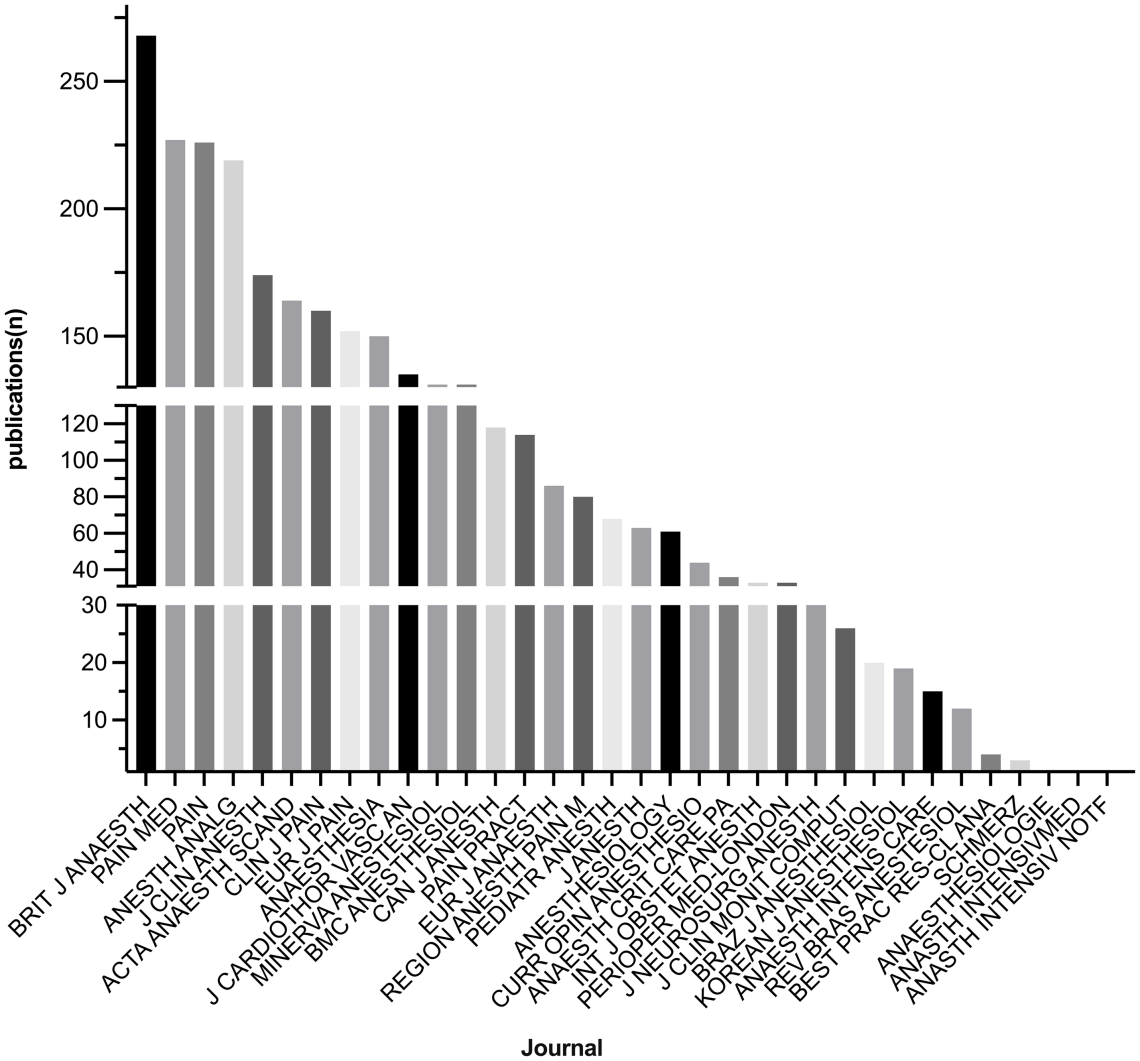
Publications of SRMAs per journal.

The total number of SRMAs published by each journal from 2013 to 2023 ranges from 0 to 268. The top 10 journals with the highest number of publications: BRIT J ANAESTH: 268/8.92%, PAIN MED: 227/7.56%, PAIN: 226/7.52%, ANESTH ANALG: 219/7.29%, J CLIN ANESTH: 174/5.79%, ACTA ANAESTH SCAND:164/5.46%, CLIN J PAIN: 160/5.33%, EUR J PAIN:152/5.06%, ANAESTHESIA: 150/4.99%, J CARDIOTHOR VASC AN: 135/4.49%, as listed in Table 2 and Figure 2.

### 4.7 Citations

#### 4.7.1 Total citations

The total citations of the 3,004 SRMA from 2013 to 2023 were 86,145. The range of total citations for each journal was from 0 to 13,713. The top 10 journals with the highest number of total citations: BRIT J ANAESTH: 13,173/15.29%, PAIN: 12,991/15.08%, ANESTH ANALG: 8,034/9.33%, ANAESTHESIA: 5,818/6.75%, EUR J PAIN: 5,532/6.42%, CLIN J PAIN: 4,575/5.31%, PAIN MED: 4,464/5.18%, ANESTHESIOLOGY: 3,588/4.17%, J CLIN ANESTH: 3,223/3.74%, ACTA ANAESTH SCAND: 2,820/3.27%, as listed in Table 2.

#### 4.7.2 Average citations per year

Each journal’s average citations per year was from 0 to 58.82. The top 10 journals with the highest number of average citations per year: ANESTHESIOLOGY: 58.82, PAIN: 57.48, BRIT J ANAESTH: 49.15, SCHMERZ: 43.33, ANAESTHESIA: 38.79, ANESTH ANALG: 36.68, EUR J PAIN: 36.39, REGION ANESTH PAIN M: 28.81, CLIN J PAIN: 28.59, EUR J ANAESTH: 26.77, as listed in Table 2.

#### 4.7.3 Citations of more than 100 SRMAs

Out of the 34 journals, 18 of them contained a total of 160 SRMAs that received more than 100 citations each. The top 10 journals with SRMAs with more than 100 citations: BRIT J ANAESTH: 36/22.50%, PAIN: 33/20.63%, EUR J PAIN: 16/10.00%, ANESTH ANALG: 15/9.38%, ANESTHESIOLOGY: 14/8.75%, ANAESTHESIA: 9/5.63%, CLIN J PAIN: 8/5.00%, PAIN MED: 6/3.75%, ACTA ANAESTH SCAND: 4/2.50%, REGION ANESTH PAIN M: 3/1.88%, EUR J ANAESTH: 3/1.88%, PAIN PRACT: 3/1.88%, as listed in Table 2.

### 4.8 Highly cited papers

Among the 3,004 SRMAs, 36 (0.03%) of them were highly cited papers: PAIN: 15/41.67%, BRIT J ANAESTH: 9/25.00%, ANAESTHESIA 4/11.11%, ANESTH ANALG: 2/5.56%, EUR J ANAESTH: 2/5.56%, ANESTHESIOLOGY: 1/2.78%, J CLIN ANESTH: 1/2.78%, CLIN J PAIN: 1/2.78%, ACTA ANAESTH SCAND: 1/2.78%, as listed in Supplementary 4.

Out of the 36 highly cited papers, 22 focused on “pain management.” Out of the 22 papers focused on “pain management,” 5 were focused on chronic pain, 8 were focused on perioperative pain, one of which was specifically about perioperative pain in children. Additionally, 2 papers focused on neuropathic pain, 2 focused on conditioned pain, and 2 focused on physical, psychological, and self-management interventions for pain. There was a paper on instruments used for measuring non-specific low back pain, a study that examined the reliability of divergent published trial data in spinal pain, and another paper that looked into the prevalence of pain symptoms of musculoskeletal origin following coronavirus (COVID) infection, as listed in Supplementary 4.

### 4.9 Keywords

**Figure 3:**
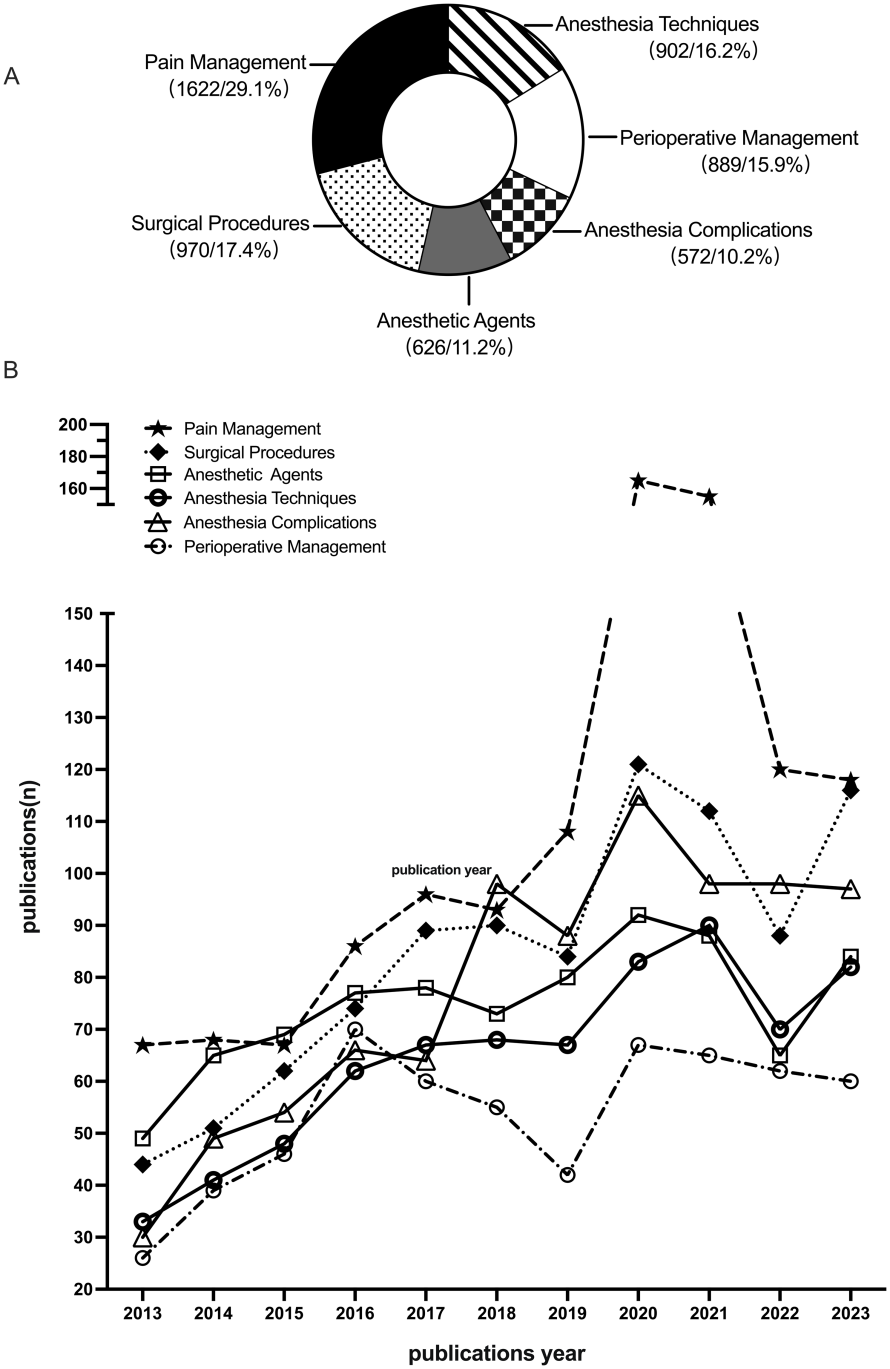
**(A)** Each topic’s keyword frequency and percentage. **(B)** Number of SRMAs per topic.

The 5,581 keywords were categorized into 6 topics, and each topic’s keyword frequency and percentage were: Pain Management (1,622/29.1%), Surgical Procedures (970/17.4%), Anesthesia Techniques (902/16.2%), Perioperative Management (889/15.9%), Anesthetic Agents (626/11.2%), Anesthesia Complications (572/10.2%), as shown in Figure 3A and listed in Table 3.

**Table 3:** Keywords classification.

Topic	Frequency/percentage
Anesthesia Techniques	902/16.2%
regional anesthesia	199
general anesthesia	175
nerve block	105
neuraxial anesthesia	81
lumboabdominal nerve block	67
upper limbs block	42
lower limbs block	39
thoracic nerve block	29
Anesthesia Management	889/15.9%
airway management and mechanical ventilation	278
patient safety	237
organ perfusion	175
fluid therapy	82
recovery	81
patient blood management	36
Anesthesia Complications	572/10.2%
complication	249
postoperative cognitive dysfunction	168
acute kidney injury	66
postoperative nausea and vomiting	47
acute lung injury	27
hoarseness	8
cerebrospinal injury	7
Surgical Procedures	970/17.4%
surgery	352
cardiac surgery	263
abdominal surgery	98
orthopedic surgery	97
obstetric operation (cesarean section)	85
breast surgery	42
thoracic surgery	20
urologic surgery (prostatectomy)	8
thrombectomy	3
gynecological surgery (hysterectomy)	2
Anesthetics	626/11.2%
opioids	169
local anesthetics	97
propofol	86
volatile anesthetics	84
corticosteroid	62
midazolam	28
nonsteroidal anti-inflammatory drugs	23
clonidine	20
nitrous oxide	17
ketamine	11
pregabalin	9
ondansetron	5
tramadol	4
magnesium sulfate	3
gabapentin	3
epsilon aminocaproic acid	3
amitriptyline	2
Pain or Pain Management	1622/29.1%
pain management	423
chronic pain	303
postoperative pain	229
low back pain	196
acute pain	179
neuropathic pain	104
musculoskeletal pain	40
knee osteoarthritis	35
back pain	30
osteoarthritis	21
neck pain	15
cancer pain	11
headache	9
rheumatoid arthritis	9
shoulder pain	7
phantom limb pain	3
hyperalgesia	3

Number of SRMAs per topic was as follows: “Pain Management” had 1,143 SRMAs, “Surgical Procedures” had 931 SRMAs, “Anesthetic Agents” had 823 SRMAs, “Anesthesia Techniques” had 707 SRMAs, “Perioperative Management” had 595 SRMAs, “Anesthesia Complications” had 853 SRMAs, as shown in Figure 3B.

## 5 Discussion

The present study comprehensively analyzed SRMA in indexed anesthesia journals from 2013 to 2023, including their JCR partition, IF, H-index, total/annual number of publications, citations, institutions, and countries that published the most literature. Furthermore, they helped researchers or clinicians to master the research trends precisely and quickly, thereby aiding in conducting further studies.

The BRIT J ANAESTH had the highest 5-year impact factor (9.6), the greatest number of publications (268/3,004), the highest total number of citations (13,173/86,145), and the greatest number of SRMAs cited more than 100 (36/160). The ANAESTHESIA achieved the highest IF in the 2022 JCR (IF 10.7) and the highest average yearly citations (58.82). The PAIN had the highest number of highly cited papers (15/36). The United States was the most productive country, with 823/3,004 SRMAs. The University of Toronto (Canada) had the highest number of publications (245/3,004).

This study includes publications only in English since it is the international language of science (11). ISI and WoS databases mainly include English journals, which makes it difficult for journals in other languages to attain high impact (12). Dogan and Karaca reviewed anesthesia-related publications that were indexed in the WOS between 2009 and 2018; the USA produced more than one-fourth (28.9%) of the literature, with 89.7% of the documents published in English (13).

According to the 2022 JCR, 34 anesthesia journals were included in our study, while only 18 anesthesia journals were listed in the 2007 JCR (2). The number of anesthesia-related journals has nearly doubled in the past 15 years. The IF of the 18 journals ranged from 0.28 to 4.6 in the 2007 JCR, with none having an IF greater than 5. However, the average 5y-IF of the 34 anesthesia journals in 2022 JCR ranged from 0.5 to 9.6. Among these were 7 (21.00%) journals with an IF greater than 5.

The IF is a metric that calculates the average number of citations received by SRMAs published in a journal. If the IF of a journal is 5, it means that, on average, each SRMA published in the journal receives five citations within the first 2 years. In 2008, the Thomson Institute for Scientific Information introduced the 5y-IF, besides the conventional 2-year IF (2y-IF). The 5y-IF might provide a more accurate representation of a journal’s quality over the last 10 years. The IF was created to evaluate a journal’s quality, not individual SRMAs’ quality (14). However, authors and readers should be aware of several caveats (9, 15, 16): IF is an average, a few highly cited SRMAs can easily skew it. IF is calculated over a two-year window and does not consider later spikes in citations to an SRMA. Several communities benefit from reading SRMAs even if they do not write or cite them. A journal with a low IF may still contain valuable content that impacts readers. IF varies depending on the type of SRMA. Review SRMAs are often cited more frequently, resulting in journals that publish them having higher IF. The IF only considers citations from a limited number of indexed journals and does not consider citations from non-indexed journals.

The citations to an article are highly related to the quality of the article and the novelty of the findings. Importantly, citation counts are influenced by various factors (17). Some factors like structured abstracts (18) and study design (19) may reflect the reporting quality and strength of evidence. On the other hand, visibility and accessibility may be affected by factors such as open access (20) and title length (21). The relationship between citation counts and quality must be considered carefully due to citation bias, the preferential to cite statistically significant results that may inflate efficacy expectations (22). Certain studies may be cited more often if published in high-reputation journals despite similar quality to those in lesser-known journals. As this was a bibliometric study, we did not review the quality and reporting of meta-analyses, and these considerations have been addressed elsewhere (23). It is important to note that citation counts can be useful in measuring the level of interest in anesthesia-related research within the research community. However, it is essential to understand that these metrics may not accurately reflect the clinical effectiveness of these modalities or the practicality of implementing new guidelines in clinical practice.

In the present study, the annual number of SRMA increased from 2013 to 2021. The minimum number of SRMAs recorded was 145 in 2013, with a peak of 385 in 2021. The number of articles published in a particular medical field measures its productivity (24). Publications share knowledge and new findings and increase an author’s recognition within the medical community. It may enable more accessible access to research funding for future studies (25). The number of original articles published by a country or institution indicates their contribution to creating new knowledge in anesthesia. The strength of this metric lies in its intrinsic validity, as opposed to surrogates such as the number of academic staff and independent grant funding, which can be affected by factors such as cost-effectiveness (2, 26). Studies found that high-income countries, particularly the USA, produced most global anesthesia publications analyzed in highly cited journals (2, 27). From 2000 to 2009, middle-income countries, particularly Turkey, China, and India, published more articles than a decade earlier (9, 14). A decline in the original research published by the USA (28), the United Kingdom (26), and Canada (4) in anesthesia journals was observed between 1997 and 2008.

Anesthesia is a broad topic, and many anesthesia-related articles have been published in non-anesthesiology field journals (9, 28). Various reasons explain the significant decline in surgical research in high-income countries. One of the reasons is the increased pressure to generate funds through clinical practice. Additionally, there are increasingly stringent institutional review board requirements, making obtaining informed consent and eligibility for research difficult. Another reason is the lack of governmental and private funding (4, 28). The shortage of anesthesiologists worldwide has also impacted research activities. The demands of clinical duties have reduced the time available for research activities (4, 28). According to a survey conducted by the Society of Academic Anesthesiology Chairs/Association of Anesthesiology Program Directors in August 2000, 91.5% of academic departments required additional anesthesiologists, while 66.5% of necessary departments additional coverage by certified registered nurse anesthetists. Academic positions in anesthesiology have remained unfilled, and those practicing at academic centers are forced to spend more time performing clinical duties. Consequently, they have less time to devote to teaching and research activities (4, 28).

In the present study, Canadian institutions accounted for 30% of the top 10 institutions with the highest number of articles, with the University of Toronto having the highest number of publications, listed in Supplementary 3. Our results are consistent with the research of Tsui et al.; from 2000 to 2004, the University of Toronto had the highest number of publications (4). Total Canadian anesthesia publications remained constant from 2000 to 2004. The number of randomized controlled trials (RCTs) conducted seems to be decreasing, whereas the number of case reports and reviews published has remained constant over the past 5 years. The University of Toronto had the highest number of publications in this five-year time frame. These universities conducted primarily RCTs, whereas smaller Canadian universities mainly published case reports, reviews, and cohort studies (4). The University of Toronto has the largest Canadian anesthesia residency program in the number of teaching staff, hospital facilities, and patient volume. Such resources offer an unequaled opportunity for University of Toronto anesthesia residents and staff to provide a supportive environment for learning and inquiry in all aspects of anesthesia. The University of Toronto is the leader of Canadian anesthesia departments in terms of research productivity (4).

Previous reports confirm a decline in anesthesia publications, particularly original articles and RCTs from high-income countries (4, 26, 28). In the present study, from 2013 to 2023, high-income countries, particularly the USA, produced the most significant number of SRMA in indexed anesthesia journals. The USA had the highest number of SRMA (823/3,004; 27.40%), followed by Canada (513/3,004; 15.35%), and United Kingdom (461/3,004; 15.35%) 461, as listed in Supplementary 2.

RCTs are the most reliable study design to answer a specific clinical question, making them a cornerstone of evidence-based medicine. Performing RCTs with a large sample size is a challenge for researchers in reaching reliable statistical outcomes (29). Systematic reviews and meta-analyses allow combining data from individual RCTs to reach more robust and reliable conclusions regarding a clinical question. SRMA is a statistical method that provides objective and quantitative estimates on a specific topic. Understanding how to conduct a meta-analysis is useful for clinicians in making clinical decisions (30). Furthermore, compared to RCTs, meta-analyses do not require additional funding, ethics committee approval, qualifications, consent signing, or sample collection/testing. The results of meta-analyses can serve as academic achievements for authors, aiding in the advancement of their careers, obtaining financial support to conduct RCTS, and saving resources (manpower and money).

In the present study, keywords related to anesthetic agents were mainly traditional anesthetics, such as opioids, local anesthetics, propofol, and volatile anesthetics. Some newer anesthetics, such as ciprofol correlated SRMA, did not appear in the 34 indexed anesthesia journals between 2013 and 2023, while ciprofol (31, 32) correlated meta-analyses appeared in indexed anesthesia journals in 2024. In the topic of surgical procedures, there were almost no operations related to large arteries, such as the aorta, pulmonary artery, or vena cava surgery.

Among the 3,004 SRMAs, 36 (0.03%) SRMAs were highly cited papers; out of the 36 highly cited papers, 22 focused on “pain management.” Opioids are administered peri-operatively for postoperative analgesia and intra-operatively to control sympathetic responses to surgical stimuli, frequently as a surrogate for presumed pain (33). As there is strong evidence that opioid-inclusive anesthesia does not reduce postoperative pain but is associated with more postoperative nausea and vomiting when compared with opioid-free anesthesia, it suggested that anesthetists should reconsider their intra-operative opioid choices on a case-by-case basis (33).

It remains unclear whether the risks of opioid use outweigh the potential benefits in the long-term management of chronic pain (34–36). Concerns about inappropriate opioid use have grown as the incidence and prevalence of long-term opioid prescribing for chronic pain have increased. Chronic pain patients prescribed opioids is a vexing problem that is compounded by complex clinical presentations that often include mental health and persistent pain problems (34, 36).

Neuropathic pain is widely recognized as one of the most difficult pain syndromes to manage, and outcomes often are unsatisfactory (37, 38). This is partly because the contribution of neuropathy to pain presenting in primary care may be unrecognized (39), and there is evidence of suboptimal drug use in the treatment of neuropathic pain (40). Epidemiological research in this area can be problematic, and the reasons for this are multifactorial: the lack of agreed, valid case definitions that truly reflect the condition under consideration and that are feasible to apply in population-based studies; heterogeneous studies of variable quality, using different means of case ascertainment; and inclusion or exclusion of cases in which pain is not a primary presenting complaint (39).

In this study, multiple factors are related to the research results. The selection criteria for journals impact the scope and nature of the research data. We selected journals from the “Anesthesiology” category in the 2022 JCR and further screened for those with an ISSN (print) number and included them in the Science Citation Index Expanded (SCIE) of the WoS Core Collection. This ensured the standardization of the data source, but it may have excluded some valuable journals that were not included. The data retrieval was sourced from the Web of Science database, which may have missed relevant literature in other important databases. Moreover, the search keyword “anesthesia” was limited to standard American English vocabulary, potentially overlooking related studies using different expressions. At the same time, the citation situation of the literature is affected by various factors, such as article structure, research design, open-access status, and title length. These factors were not deeply explored in this study but can influence the influence of journals and articles measured by citation counts, thus affecting the research results.

The findings of this study can provide valuable insights for anesthesiology practitioners and clinicians. By leveraging the journal indicators from this study, they can strategically select high-impact journals for publication, enhancing their academic influence. Additionally, the high-frequency keywords and highly cited paper topics identified in this research can serve as a guide for future studies, helping researchers to stay at the forefront of academic trends. This study offers a comprehensive overview of the current status and hotspots in anesthesiology research and can help students make topic selection and literature review more targeted and efficient. Moreover, recognizing the limitations of this study can foster critical thinking, which is essential for conducting independent research in the future.

As the finding of the current study, the high-cited papers on pain management can directly benefit patient care. For instance, studies on different types of pain like chronic pain, peri-operative pain, and neuropathic pain offer evidence-based strategies for pain assessment and treatment. The research on using virtual reality to reduce pain and anxiety in pediatric patients during medical procedures can be directly applied in pediatric care settings. By implementing these findings, healthcare providers can improve the quality of pain management for patients, enhancing their overall experience and recovery. The study’s findings on anesthetic agents can inform clinical anesthesiologists. Although most of the research is currently on traditional anesthetics, with the emergence of new agents, the research direction may change. Clinicians can stay updated on these trends to make more informed decisions about anesthetic choices.

## 6 Limitations

Although the selected journals belong to the JCR anesthesiology category, some cover disciplines beyond anesthesiology and perioperative medicine. On the other hand, some general medicine journals may also publish a few SRMAs related to anesthesiology research. However, it is worth noting that many journals in the field of anesthesiology solely focus on pain research, which can result in a distorted assessment of the anesthesia literature. Despite this, the 34 journals included in these analyses are the indexed international journals dedicated to anesthesiology research. The study only analyzes the WoS database, which could exclude important SRMAs not indexed by WoS. However, WoS claims to provide quality literature. Another limitation was the keyword ‘Anesthesia’ which was used in the search query and was restricted to standard American (US English) vocabulary. Authors who primarily produce reviews and meta-analyses may not be equally prolific in conducting other types of studies, such as RCTs. The impact of self-citations—both by authors and journals—should be considered when evaluating the IF. A series of reviews authored by the same individual, particularly an opinion leader in a specific research area, could skew the results of a purely descriptive statistical analysis. During the analysis process, the keyword classification and topic coding were manually completed by two authors, which may introduce certain subjectivity.

## 7 Conclusion

The current study demonstrated that PAIN had the highest number of highly-cited papers. The United States was the most productive country. University of Toronto had the highest number of publications. The most frequent of keywords was the topic “Pain Management.” This present study would be valuable to practitioners, academics, researchers, and students in understanding the dynamics of progress in anesthesiology.

## Supplementary material

SUPPLEMENTARY 1 Search strategy of the Web of Science.

SUPPLEMENTARY 2 The top 10 countries with the highest number of publications.

SUPPLEMENTARY 3 The top 10 institutions with the highest number of publications.

SUPPLEMENTARY 4 Highly cited papers.
