# Supplementary material for: Publications of systematic review and meta-analysis in the indexed anesthesia journals: a 10-year bibliometric analysis
Source: Front Med (Lausanne). 2025 May 7;12:1523630. doi: 10.3389/fmed.2025.1523630 (PMC12092347; doi:10.3389/fmed.2025.1523630)
Supplement: SUPPLEMENTARY 1 — Search strategy of the Web of Science. [file Table_1.DOCX]

**Search strategy of the Web of Science**

(((((((((((((((((((((((((((((((((SO=(Anaesthesia Critical Care & Pain Medicine)) OR SO=(Best Practice & Research-Clinical Anaesthesiology)) OR SO=(JOURNAL OF NEUROSURGICAL ANESTHESIOLOGY)) OR SO=(Minerva Anestesiologica)) OR SO=(PAIN MEDICINE)) OR SO=(Korean Journal of Anesthesiology)) OR SO=(CLINICAL JOURNAL OF PAIN)) OR SO=(INTERNATIONAL JOURNAL OF OBSTETRIC ANESTHESIA)) OR SO=(Journal of Anesthesia)) OR SO=(JOURNAL OF CARDIOTHORACIC AND VASCULAR ANESTHESIA)) OR SO=(Perioperative Medicine)) OR SO=(Pain Practice)) OR SO=(Current Opinion in Anesthesiology)) OR SO=(BMC Anesthesiology)) OR SO=(JOURNAL OF CLINICAL MONITORING AND COMPUTING)) OR SO=(ACTA ANAESTHESIOLOGICA SCANDINAVICA)) OR SO=(PEDIATRIC ANESTHESIA)) OR SO=(ANAESTHESIA AND INTENSIVE CARE)) OR SO=(Brazilian Journal of Anesthesiology)) OR SO=(Anaesthesiologie)) OR SO=(SCHMERZ)) OR SO=(REVISTA BRASILEIRA DE ANESTESIOLOGIA)) OR SO=(ANASTHESIOLOGIE & INTENSIVMEDIZIN)) OR SO=(ANASTHESIOLOGIE INTENSIVMEDIZIN NOTFALLMEDIZIN SCHMERZTHERAPIE)) OR SO=(British Journal of Anaesthesia)) OR SO=(Anesthesiology)) OR SO=(REGIONAL ANESTHESIA "AND" PAIN MEDICINE)) OR SO=(Journal of Clinical Anesthesia)) OR SO=(ANAESTHESIA)) OR SO=(Pain)) OR SO=(EUROPEAN JOURNAL OF ANAESTHESIOLOGY)) OR SO=(ANESTHESIA "AND" ANALGESIA)) OR SO=(CANADIAN JOURNAL OF ANESTHESIA JOURNAL CANADIEN D ANESTHESIE)) OR SO=(EUROPEAN JOURNAL OF PAIN) AND TS = (“meta analysis” OR “meta analyses” OR “systematic review” OR “systematic reviews”), time span= “from 2013-01-01 to 2023-12-31” language = “English” AND type = “article OR review OR early access OR Proeccding paper.”
