# Supplementary material for: Publications of systematic review and meta-analysis in the indexed anesthesia journals: a 10-year bibliometric analysis
Source: Front Med (Lausanne). 2025 May 7;12:1523630. doi: 10.3389/fmed.2025.1523630 (PMC12092347; doi:10.3389/fmed.2025.1523630)
Supplement: SUPPLEMENTARY 2 — The top 10 countries with the highest number of publications. [file Table_2.DOCX]

**Supplement 2.** The top 10 countries with the highest number of publications.

|  | **Country** | **Articles/percent** |
| --- | --- | --- |
| 1 | United States | 823/27.40% |
| 2 | Canada | 513/17.08% |
| 3 | United Kingdom | 461/15.35% |
| 4 | China | 455/15.15% |
| 5 | Australia | 310/10.32% |
| 6 | Denmark | 247/8.22% |
| 7 | Germany | 214/7.12% |
| 8 | Italy | 210/6.99% |
| 9 | Netherlands | 204/6.79% |
| 10 | Switzerland | 141/4.69% |
