# Supplementary material for: Publications of systematic review and meta-analysis in the indexed anesthesia journals: a 10-year bibliometric analysis
Source: Front Med (Lausanne). 2025 May 7;12:1523630. doi: 10.3389/fmed.2025.1523630 (PMC12092347; doi:10.3389/fmed.2025.1523630)
Supplement: SUPPLEMENTARY 3 — The top 10 institutions with the highest number of publications. [file Table_3.DOCX]

|  | **Institution** | **Country** | **Articles/percent** |
| --- | --- | --- | --- |
| 1 | University Toronto | Canada | 245/8.16% |
| 2 | University Copenhagen | Denmark | 92/3.06% |
| 3 | McMaster University | Canada | 87/2.90% |
| 4 | University Ottawa | Canada | 85/2.83% |
| 5 | Copenhagen University | Denmark | 68/2.26% |
| 6 | Stanford University | United States | 68/2.26% |
| 7 | University Sydney | Australia | 68/2.26% |
| 8 | Monash University | Australia | 65/2.16% |
| 9 | University Washington | United States | 59/1.96% |
| 10 | Kings Coll London | United Kingdom | 57/1.90% |

**Supplement 3**. The top 10 institutions with the highest number of publications.
